# Composition and Antifungal Activity of the Alkaloidal Fraction of *Lupinus mirabilis* Leaves: A Biochemometrics-Based Exploration

**DOI:** 10.3390/molecules27092832

**Published:** 2022-04-29

**Authors:** Freddy A. Bernal, Ericsson Coy-Barrera

**Affiliations:** Bioorganic Chemistry Laboratory, Facultad de Ciencias Básicas y Aplicadas, Universidad Militar Nueva Granada, Cajicá 250247, Colombia

**Keywords:** *Lupinus mirabilis*, quinolizidines, alkaloids, *Fusarium oxysporum*, ultrasound-assisted extraction, antifungal activity, biochemometrics

## Abstract

*Lupinus* plants are well-recognized due to their significant alkaloid content, which has made them the subject of several studies. However, the lack of chemical and biological information on the Colombian *Lupinus* species remains a fact. Therefore, the alkaloidal fractions from the leaves of L. *mirabilis* obtained by conventional solvent and ultrasound-assisted extraction (CSE and UAE, respectively) at different time frames were analyzed. Sparteine (**2**) was the main component in all cases; however, its relative abundance showed large variability, ranging from 64.7% to 80.6%. Minor constituents were also affected by the extraction conditions. In general, prolonged times gave a higher proportion of alkaloids under CSE, while only a slight decrease was observed under UAE. Both the method and extraction time appeared to equally affect the ratios of particular alkaloids, leading to variations in their effect on the mycelial growth of *Fusarium oxysporum*. Holistic analysis through multiple-covariate statistical methods as an approach to integrating chemical and bioactivity datasets allowed inferring the compounds most likely responsible for the changes in mycelial growth inhibition. 13α-Hydroxylupanine (**12**) might represent a promising compound to be included in further studies against this phytopathogen.

## 1. Introduction

The Fabaceae family is widely distributed worldwide and is recognized by its invasive ability and the high content of specialized metabolites with multi-directional activities. One of the most abundant genera within this family is *Lupinus*, accounting for more than 260 species [1]. Plants belonging to the genus *Lupinus* are found all over the world; however, most of them grow wild in the New World [2,3]. However, and despite their significant representation, only four species have been extensively studied: *L. albus*, *L. angustifolius*, *L. luteus*, and *L. mutabilis* (commonly known as white, blue, yellow, and pearl lupine, respectively), due to their potential economic relevance as a cheap alternative to other legume crops [4]. The commercialization of *Lupinus* and lupine-derived food products [4,5] is still limited owing to the presence of variable amounts of alkaloids in all plant parts [6,7], being sometimes the main phytoconstituents causing a bitter taste of such products and even responsible for acute anticholinergic toxicity [8,9]. Thus, alkaloid-containing lupine plant materials for animal and human intake require a suitable debittering preprocessing to remove undesirable alkaloids [10].

An important diversity of alkaloids (particularly quinolizidines) is produced by *Lupinus* plants, predominantly in leaves, which are subsequently translocated to different organs, and mainly accumulated in seeds [11]. The structural patterns of quinolizidine alkaloids involve bicyclic, tricyclic, and tetracyclic moieties, whose occurrence and regulation are influenced by genotype, soil and environmental conditions, and pest and phytopathogen pressures [12]. Indeed, a defensive strategy of *Lupinus* plants against biotic factors is constituted by the production and accumulation of quinolizidine-like alkaloids [13]. Hence, quinolizidine sources can be investigated to explore their bioactivity against fungal pathogens. In this context, some fungal phytopathogens, such as *Sclerotium rolfsii*, *Alternaria solani*, *Rhizoctonia solani*, *Fusarium oxysporum*, and *F. verticillioides*, have been inhibited by quinolizidine-rich extracts from *Lupinus exaltatus, L. mexicanus,* and *L. albescens,* comprising growth inhibition > 72% and IC_50_ < 30 µg/mL [14,15].

*Lupinus* plants are also easily adaptable as they are found on dry hilly grasslands, coastal sands, cliffs, and along the banks of streams and rivers that are not suitable to many other plants [16]. In fact, several *Lupinus* species grow under such conditions in different regions of Colombia. One example is *L. mirabilis*, a lupine extensively distributed along the Bogotá plateau, mainly in highland ecosystems. Even though this species has a wide representation, there are no reports on the chemical/alkaloidal composition of wild specimens. Therefore, quinolizidine-oriented phytochemical studies on an unreported *Lupinus* plant are required to expand the knowledge on *Lupinus* chemodiversity. Such exploration becomes an opportunity to find bioactives against fungal phytopathogens, e.g., *F. oxysporum*, a relevant problem in commercial crops worldwide [17].

There are several approaches to discovering bioactives from natural sources [18]. The most common strategies are high-throughput screening (i.e., the evaluation of libraries of previously-isolated compounds) and bioguided fractionation (i.e., the consecutive evaluation of fractions from an active parent extract) to achieve final identification of the active components. However, these are time-consuming and expensive [19]. These disadvantages can be overcome by integrating chemical and bioactivity datasets to recognize plausible bioactives within plant extracts or fractions using multiple-covariate statistical methods, which recently emerged and are called biochemometrics [20,21,22]. This integration can exploit diverse extraction protocols to provide different chemical profiles that can be associated with bioactivity and, consequently, recognize statistically putative bioactives [23]. The incorporation of multiple profiles from distinct extraction conditions (e.g., time, method, etc.) and bioactivity data into a single analysis is the main benefit of this kind of approaches. They might be especially useful considering that certain extraction conditions might lead to better extracts in terms of specific target properties (e.g., bioactivity) since active principles can be differentially removed from the plant matrix. Therefore, they can be used as primary initiatives to direct the search for bioactive components from plant sources [24].

Hence, as part of our phytochemical research on highland-occurring Colombian plants, we present the GC-MS-based analysis of alkaloidal fractions from the leaves of *L. mirabilis* obtained by conventional and ultrasound-assisted extraction using different time frames as the input chemical dataset. Comparison of the bioactivity of those fractions against *F. oxysporum* and the analysis of the effect of the composition on the mycelial growth inhibition through multiple-factor statistical integration as a biochemometrics-based exploration are also shown.

## 2. Results and Discussion

### 2.1. Analysis of Alkaloidal Composition

**Figure A1:**
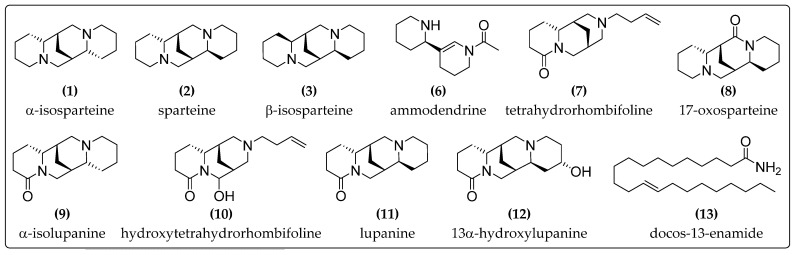
Structures of compounds identified from alkaloidal fractions of *Lupinus mirabilis* leaves.

The genus *Lupinus* is well-known for high concentrations of isoflavones [4] and quinolizidine alkaloids [6,7,8]. The latter specialized metabolites are especially important to *Lupinus* as chemotaxonomic markers. The relevance of this kind of compound for ecological interactions and nitrogen-storing processes has been described previously [25]. In the present study, the composition of the alkaloidal fractions from *L. mirabilis* leaves, obtained by two extraction methods, namely conventional solvent extraction (CSE) and ultrasound-assisted extraction (UAE), during three different extraction periods (1–3) is described. The identified nitrogen-containing compounds along with key chromatographic and spectrometric data by GC-MS [26] are listed in Appendix A Table A1 and their structures are depicted in Appendix A Figure A1. These compounds have been previously described in *Lupinus* species and are therefore considered lupine alkaloids [6,7,27]. They account for different compound classes as follows: tetracyclic quinolizidine alkaloids (**1**–**3**, **8**, **9**, **11**, and **12**), tricyclic quinolizidine alkaloids with allylic lateral chain (**7** and **10**) [28], and dipiperidines **6**) [6]. The identity of each compound was confirmed by a thorough comparison of representative MS signals and RI values with those reported in the literature (Appendix A Table A1) [27,29].

To study the effect of both extraction method and extraction duration on the resulting alkaloidal composition, we assessed three different time frames for each method. Table 1 shows the relative percentage of each nitrogen-containing compound in all the analyzed samples. Two abundant compounds consistently appeared: sparteine (**2**) and lupanine (**11**). Although both alkaloids are typically found in *Lupinus*, in most cases, quinolizidine **11** has been reported as the main constituent [27,30,31,32,33]. Wink et al. [9] found **2** in a higher percentage than **11** only in eleven out of 109 accessions of *Lupinus* around the world, accounting for 56 different species. *L. arboreus* (seeds and leaves), *L. arcticus* ssp. *subalpinus* (leaves), *L. sericeus* ssp. *huffmannii* (leaves), and *L. sericeus* ssp. *flexuosus* (leaves) contained exceptionally high levels of **2** (>70% of the alkaloidal fraction) [9]. More recently, Kordan et al. [33] reported three samples of *L. luteus* (L. cv. Dukat, Perkoz, and Talar) with higher amounts of **2** than **11**. Such a study was performed on twelve lupine accessions, including four different species. On the other hand, the existence of chemotypes within *Lupinus* species has also been described [32,33,34]. For instance, one out of seven chemotypes observed in *L. sulphureus* displayed exclusively a **2**/**11** ratio above the unit (in 49 accessions analyzed) [34]. Such diversity encouraged us to further analyze the Colombian *L. mirabilis* specimens. Recently, we studied the alkaloidal profile of a greenhouse-propagated specimen of *L. mirabilis* and a high **2**/**11** ratio (>1.6) was observed [35].

The alkaloidal composition suffered changes upon variation of the extraction conditions, including even the complete absence of some of the minor constituents in some cases (Table 1). A detailed examination of Table 1 establishes that CSE1 has a significantly lower percentage of alkaloids than the other fractions (*p* < 0.05). This indicates that short extraction periods are unsuitable for the selective extraction of alkaloids leading to more complex mixtures containing terpenoids (as evidenced by GC-MS; data not shown). In contrast, shorter periods using UAE afforded a more significant proportion of nitrogen-containing compounds overall (*p* < 0.05). In addition, an extension of the extraction period did not show substantial changes in the total proportion of alkaloids; however, individual variations were observed (e.g., UAE1 vs UAE3).

**Figure 1:**
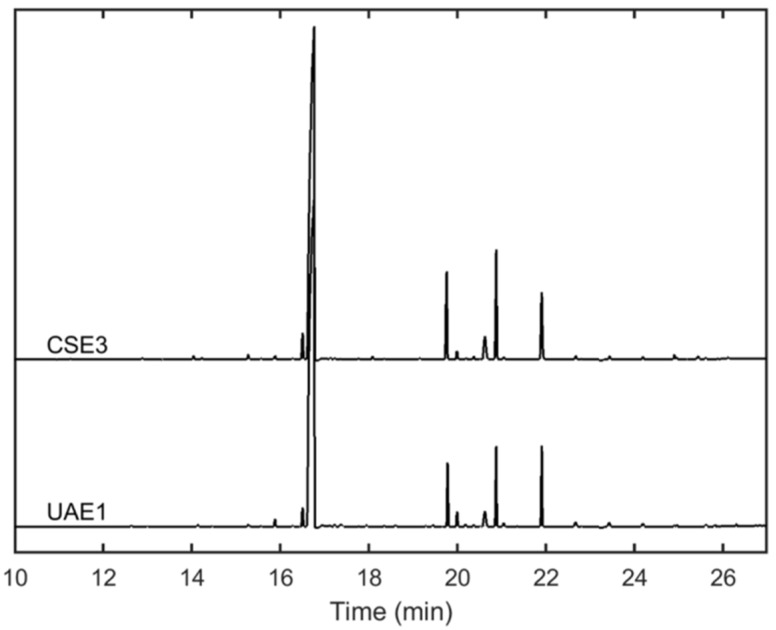
Comparison of the GC-MS alkaloidal profiles of *L. mirabilis* leaves obtained by conventional extraction for 6 h (CSE3) and ultrasound-assisted extraction for 0.5 h (UAE1).

From Table 1, it is evident that the ratio **2**/**11** changed along with the variation of the extraction time (from 16.4 to 10.5 and from 7.9 to 5.3 for CSE and UAE, respectively). While longer extraction times under CSE afforded a significantly higher proportion of both compounds (*p* < 0.05), increasing the contact period under UAE caused a substantial decrease in the relative amount of **2** with an imperceptible variation of the ratio of **11**. Other compounds (such as **7** and **12**) were increasingly concentrated over time regardless of the extraction method. Interestingly, CSE3 and UAE1 exhibited the closest similarity in terms of their alkaloidal composition (Table 1). The GC-MS profiles for these two samples are almost indistinguishable as demonstrated in Figure 1.

**Figure 2:**
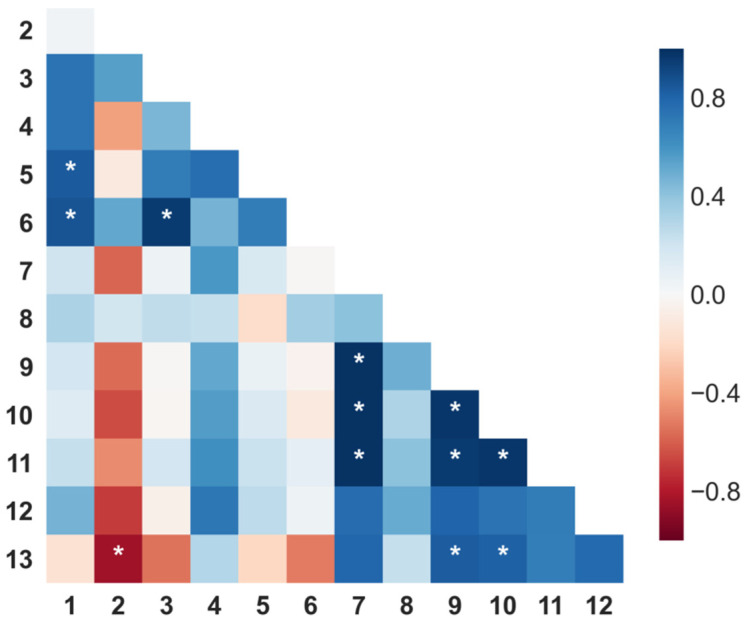
Pearson’s correlations among alkaloids of *L. mirabilis* from different extraction conditions. Dark blue: high positive correlation; dark red: high negative correlation. * Significant *p* value (*p* < 0.05). **1**: α-isosparteine; **2**: sparteine; **3**: β-isosparteine; **4** and **5**: unknown; **6**: ammodendrine; **7**: tetrahydrorhombifoline; **8**: 17-oxysparteine; **9**: α-isolupanine; **10**: hydroxytetrahydrorhombifoline; **11**: lupanine; **12**: 13α-hydroxylupanine; and **13**: 13-Docosenamide.

Pearson’s correlation coefficients from the relative proportion of alkaloids among samples were calculated (Figure 2) while looking for general insights. In agreement with the observable trends from Table 1, several alkaloids seemed to be somewhat positively correlating with coefficients above 0.8 (Figure 2). However, the statistical significance of most of these was considerably low as indicated by the correspondingly high *p* values (only *p* < 0.05 are indicative of statistical significance and therefore marked with a star). Interestingly, a high positive significant correlation between **7** and **9**, **10**, and **11** was found. This finding could indicate that the extraction method and the variation in the exposure time affect the amount of those four alkaloids in the same way. Therefore, their ratio rather than the relative proportion in the alkaloidal mixture remained almost unchanged.

### 2.2. Mycelial Growth Inhibition

The effect of the alkaloidal fractions on the mycelial growth of the *F. oxysporum* LQB-03 strain was also studied. The results are presented in Table 2. As can be seen, all the samples were found to be active at different levels, comprising IC_50_ values within the range of 33.5–98.5 µg/mL, although less potent than the positive controls used (i.e., prochloraz IC_50_ = 17.7 µg/mL, and mancozeb IC_50_ = 11.9 µg/mL). This finding agrees with previous studies, where alkaloid extracts have shown activity against phytopathogens, including *Alternaria porri*, *Aspergillus niger*, *Monilia fructicola*, and *F. oxysporum* [36,37,38]. Moreover, both types of extraction (i.e., CSE and UAE) led to similar trends (the longer the extraction time, the higher the activity). Particularly, UAE resulted as the most suitable extraction procedure to conveniently enrich the fractions in active alkaloids (86.3 µg/mL < IC_50_ < 33.5 µg/mL), whereas CSE-derived fractions were considerably less active even after long extraction periods (98.5 µg/mL < IC_50_ < 84.8 µg/mL).

The previously reported low antifungal potential of **2** against *F. oxysporum* [37] would suggest that the observed activity for *L. mirabilis* alkaloids may be due to their minor components. Indeed, it has been described that **2** and **11** have a low-to-moderate contribution to the inhibitory action of eight *Lupinus* plants on *F. oxysporum* despite their high abundance [35]. On the other hand, and since the major constituent is seemingly inactive, the antifungal activity of quinolizidines may be the consequence of synergistic effects. Similar results have been reported for *Calia secundiflora*, whose alkaloidal fraction demonstrated activity against *F. oxysporum* [36]; however, its main component (cytisine) was characterized as a rather poor antifungal. In the present study, a reduction in the proportion of **2** and an increase for **11** and **7** are the most notorious changes among fractions (Table 2), which, in principle, might be related to the enhanced bioactivity of UAE-based fractions.

### 2.3. Variation of Chemical Composition and Mycelial Growth Inhibition by Extraction Conditions: A Biochemometrics-Based Exploration

**Figure 3:**
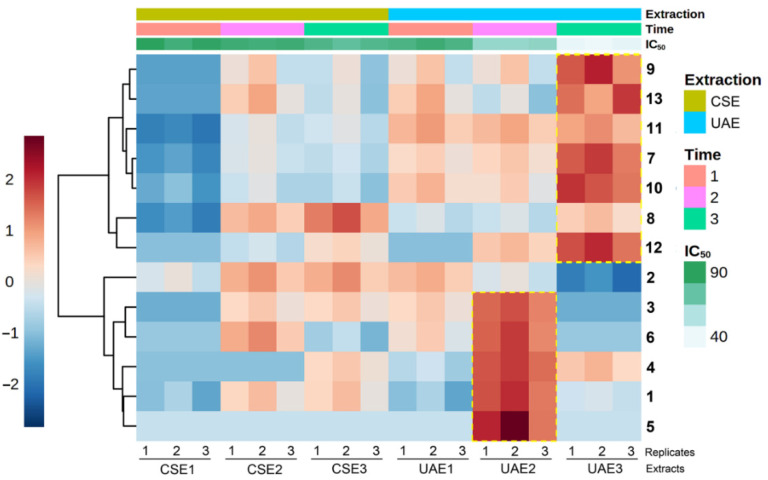
Distribution of the relative abundance of the quinolizidine alkaloids (**1**–**12**) and amide **13** detected in extracts of *L. mirabilis* under different extraction conditions. CSE1-3: conventional solvent extraction for 2, 4, and 6 h, respectively; UAE1-3: ultrasound-assisted extraction for 0.5, 1, and 2 h, respectively. The heat map is organized by columns for each factor, i.e., the extraction type (CSE and UAE), extraction time (1, 2, and 3), and mycelial growth inhibition expressed by green shades (dark green: IC_50_ = 90 µg/mL; light green: IC_50_ = 40 µg/mL). Cells colored by autoscaled relative abundance of each alkaloid (dark red: high abundance; and dark blue: low abundance). The heat map is also organized by rows according to the Ward clustering algorithm (Euclidean distance).

An integrative analysis of the alkaloidal composition and activity on *F. oxysporum* mycelial growth was performed employing multiple-covariate statistics to achieve a more general comprehension of the obtained results and infer reliable relationships. The main goal was to recognize patterns and predict plausible bioactive alkaloids within the extracts. Thus, following a suitable pretreatment and preprocessing of the GC-MS profiles, an alkaloid abundance table (AAT) was built. The overall information contained in the AAT was visualized through a heat map combined with hierarchical clustering (Figure 3). Two clusters were evidenced in this heat map, comprising compounds mainly accumulated in UAE2 and UAE3, respectively, which influenced the discrimination of mycelial growth inhibition (significantly lower IC_50_ values). Each cluster involved six different alkaloids.

**Figure 4:**
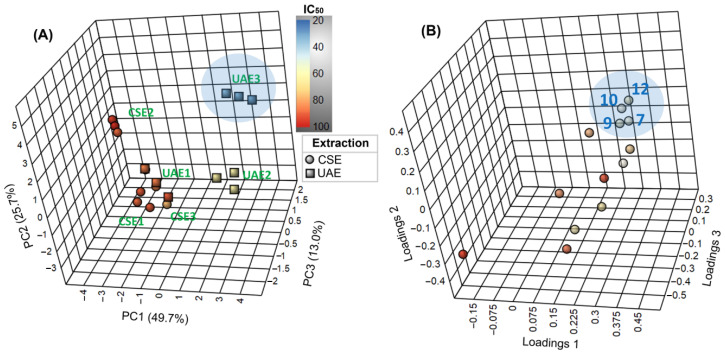
Two-factor principal component analysis (PCA) on the alkaloidal profiles of *L. mirabilis* under different extraction conditions. CSE1-3: conventional solvent extraction for 2, 4, and 6 h, respectively; UAE1-3: ultrasound-assisted extraction for 0.5, 1, and 2 h, respectively. (**A**) Three-dimensional scores plot. Samples colored by activity on *F. oxysporum* mycelial growth (expressed as IC_50_ in µg/mL). (**B**) Three-dimensional loadings plot. Compounds highlighted in blue ellipsoids are as follows: **7**: tetrahydrorhombifoline; **9**: α-isolupanine; **10**: hydroxytetrahydrorhombifoline; and **12**: 13α-hydroxylupanine.

Further analysis was performed through a two-factor principal component analysis (PCA), whose scores and loadings plots are presented in Figure 4. The first three PC’s explained 88.4% of the total variance, indicating a well-fitted model. The relatively dispersed appearance of the samples across the corresponding three-dimensional scores plot (Figure 4A) implies high diversity among the alkaloidal profiles. In particular, UAE2, UAE3, and CSE2 are located in different quadrants along the PC1-PC2 plane, indicating stronger differences in their alkaloidal compositions. 

In contrast, CSE1, CSE3, and UAE1 appeared closely related, as observed in Table 1. On the other hand, the color scale according to the IC_50_ values indicated an evident association between the alkaloidal composition and mycelial growth inhibition. The respective loadings plot (Figure 4B) revealed that four compounds were highly linked to the discriminating influence on the most active extract (i.e., UAE3), as follows: **7**, **9**, **10**, and **12**. This finding might indicate that they are the compounds responsible for the bioactivity observed for UAE3.

**Figure 5:**
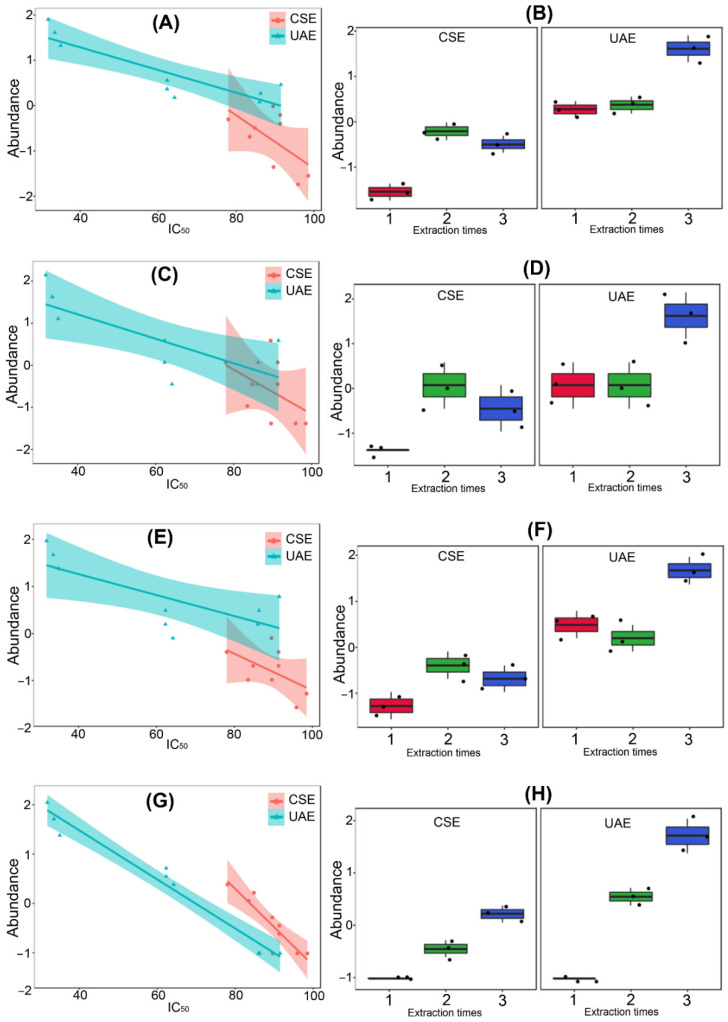
Variations of the most-contrasting alkaloids between alkaloidal profiles of *L. mirabilis* using different extraction conditions along the activity on *F. oxysporum* mycelial growth (expressed as IC_50_ in µg/mL). Linear regressions for compounds (**A**) **7**, (**C**) **9**, (**E**) **10**, and (**G**) **12** and box-plots of the autoscaled abundances for compounds (**B**) **7**, (**D**) **9**, (**F**) **10**, and (**H**) **12**. CSE1-3: conventional solvent extraction for 2, 4, and 6 h, respectively; UAE1-3: ultrasound-assisted extraction for 0.5, 1, and 2 h, respectively. Compounds: **7** = tetrahydrorhombifoline; **9** = α-isolupanine; **10** = hydroxytetrahydrorhombifoline; and **12** = 13α-hydroxylupanine.

Therefore, the variation of these compounds along factors (i.e., the extraction type, extraction time, and IC_50_ values) is presented in Figure 5. A significantly higher abundance of the four top-ranked compounds in UAE3 compared with the other fractions derived from UAE and CSE was observed, supporting their statistical influence on sample discrimination regarding bioactivity. Hence, a noticeable change along the IC_50_ values was observed for compounds **7**, **9**, and **10**, particularly in the ultrasound-assisted extraction at the longest time (i.e., UAE3), whose association between the abundance of these compounds and the activity was relevant (Figure 5A–F). However, the most drastic variations were experienced by alkaloid **12** (both by extraction type and time frame) since the slope of the linear regression is more pronounced along with IC_50_ values (Figure 5G) and involves significant differences between the extraction times for the UAE and CSE methods (Figure 5H). This observation could indicate a more plausible influence of **12** on the activity against the *F. oxysporum* strain. Highly abundant compounds, such as **2** and **11**, could not be associated with the inhibitory activity since the changes in their abundance were not statistically relevant, and they behaved as a blank.

## 3. Materials and Methods

### 3.1. Plant Material

Leaves of the plant were collected in October of 2013 near the reservoir El Hato in Usme (Bogotá, Colombia). A voucher specimen was deposited at the Colombian National Herbarium with the number COL572777. Botanist C. A. Parra identified the specimen as *Lupinus mirabilis* C. P. Sm.

### 3.2. Conventional Extraction

Air-dried, powdered material (1.0 g) was mixed with 10% HCl at 1:1 *w/v* ratio, and the resulting mixture was magnetically stirred for 2 h at room temperature. Subsequently, the residue was filtered off. Then, the filtrate was neutralized with 10% ammonia and extracted with dichloromethane (3 × 20 mL). The combined organic layers were dried over anhydrous sodium sulfate and concentrated under reduced pressure to afford the alkaloidal fraction CSE1. The same procedure was independently repeated to give alkaloidal fractions after 4 and 6 h of extraction (CSE2 and CSE3, respectively). Each extraction procedure was performed in triplicate.

### 3.3. Ultrasound-Assisted Extraction

Air-dried, powdered material (1.0 g) was mixed with 10% HCl at 1:1 *w/v* ratio. The mixture was then immersed into an ultrasonic bath for 30 min at room temperature. After that period, filtration, neutralization, and extraction were conducted as described above for the conventional extraction to afford fraction UAE1. The same procedure was independently repeated to afford alkaloidal fractions after 1 and 2 h of extraction (UAE2 and UAE3, respectively). Each extraction was performed in triplicate.

### 3.4. GC-MS Analysis

Each alkaloidal fraction was analyzed by GC-MS on a Shimadzu QP2010 Plus system (Shimadzu, Columbia, MD, USA), using a fused capillary silica column Shimadzu SHRXi 5MS (30 m × 0.25 mm; 0.25 μm coating thickness). The injection port, ionization chamber, and transfer line temperatures were 250, 230, and 325 °C, respectively. The separation was conducted by running a temperature program as follows: 70 °C (2 min), 10 °C/min up to 310 °C, and finally, the temperature was held (10 min). A quadrupole analyzer in automatic frequency scanning (full scan) was used over the mass range *m/z* 40–400. 

Mass spectra were obtained by electron ionization (EI) at 70 eV. The alkaloidal fractions were properly dissolved in chloroform before analysis and 1 μL of such solutions was injected using a split ratio of 1:10. Helium 4.5 was used as a carrier gas at a 1 mL/min flow rate. The components of each fraction were identified by comparison of their MS spectra with those reported in the database NIST95 and the literature [27,29]. Retention indexes (RI) were also calculated from a homologous series of *n*-alkanes (C_12_–C_28_) to support the identification [27].

### 3.5. Mycelial Growth Inhibition Assay

A micro-scale amended medium protocol was employed based on a previously reported procedure [39]. Briefly, six ten-fold serial dilutions (0.01–1000 µg/mL) of extracts and positive controls were prepared by amending fresh semi-solid medium (1.2 g of PDB (potato-dextrose broth) and 0.5 g of agar per 100 mL of distilled water) with the required amount of each test extract (*n* = 6). A stock dispersion was initially prepared by a direct mixture between the semi-solid medium (5 mL) and the required extract amount. This mixture was vigorously stirred until achieving a homogenous dispersion before solidifying. 

Tween-20 (5%) was used to assist the dispersion of the extracts. The resulting homogeneously amended medium was placed into three wells of a 12-well autoclavable glass plate. The final volume (for extracts and controls) was 200 μL per well (1-cm diameter). Subsequently, a 1-mm agar-mycelial plug from 5 day actively growing cultures of the *F. oxysporum* LQB-03 strain (a virulent isolate obtained from wilting *Physalis peruviana* plants [22]) was inoculated onto the center of each well. Each 12-well plate was placed into a 17-mm Petri dish, under the appropriate conditions of humidity and sterility, and sealed with plastic film. 

Each trial comprised a randomized design with three replicates for each extract compared to an absolute control (untreated, inoculated semi-solid medium). Prochloraz and Mancozeb were used as positive controls. After inoculation and sealing, this assembly was incubated at 25 °C. The assay was concluded once the colony on the negative control covered the whole well (after ca. 48 h). Therefore, the mean colony area (mm^2^) was measured for treated and untreated wells by processing the respective photographic records using the software Image J^®^ (National Institutes of Health, Bethesda, MD, USA). 

The percent mycelial growth inhibition (MGI) was then calculated for each replicate. This calculation was made employing the following equation: % MGI = [(absolute control area—treatment area)/absolute control area)] × 100. The half-maximal inhibitory concentrations (IC_50_ in μg/mL) for each extract were calculated through non-linear regression from the dose-response curves using the software GraphPad Prism 5.0 (GraphPad Software, San Diego, CA, USA).

### 3.6. Data Analysis

The data normality was checked by the Shapiro-Wilks test. Subsequently, a two-way ANOVA followed by post-hoc HSD Tukey’s test were used to assess the significant differences between relative abundances of quinolizidines (Table 1), owing to their normal distribution. On the other hand, the non-parametric Kruskal–Wallis test followed by a Dunn’s multiple comparison test were used to assess the differences among IC_50_ values (Table 2). In all cases, the significance level was set at α < 0.05. These statistical analyses were performed in R project software version 3.0.2 (R Foundation, Vienna, Austria).

On the other hand, the relative composition of each fraction was obtained after a conventional pretreatment of data. In brief, the baseline was corrected using MZmine 2 (MZmine Development Team, San Diego, CA, USA), and the resulting profiles were integrated. Thus, a data matrix was defined by the relative abundance of each alkaloid contained into the alkaloidal fractions and, therefore, an alkaloid abundance table (AAT) was built. 

First, trend variations in the alkaloid content within the samples were analyzed by direct comparison in terms of Pearson’s correlation coefficients, and the corresponding *p* values evaluated their statistical significance. This analysis was performed in MATLAB R2016a (MathWorks, Natick, MA, USA). In addition, the AAT matrix and inhibition percentages against *F. oxysporum* were also used to build a multiple-covariate input dataset, using metadata factors, i.e., extractions times and type of extraction as categorical variables, and IC_50_ values as the continuous variable. 

The resulting metadata matrix was autoscaled (mean-centered and divided by the standard deviation of each variable), visualized through a heatmap combined with hierarchical clustering, and further analyzed through two-factor principal component analysis (PCA), using the default parameters of the statistical analysis module of MetaboAnalyst 4.0 (McGill University, Quebec, Canada) [40]. The alkaloidal composition and mycelial growth inhibition were then statistically integrated, and, subsequently, the detected associations were used to explore patterns and to identify potential antifungal alkaloids within the test extracts. The variations of the most discriminating alkaloids were comparatively studied through box plots and linear regressions.

## 4. Conclusions

The alkaloidal composition of leaves from *L. mirabilis* wildly growing in Colombia was established for the first time, evaluating the effect of two different extraction methods. Regardless of the extraction conditions, sparteine (**2**) and lupanine (**11**) were identified as the main constituents of the obtained fractions, accounting for 75–89% of the total composition. This fact indicates that *L. mirabilis* is an excellent source of simple quinolizidines, such as sparteine. Tetrahydrorhombifoline (**7**), 17-oxosparteine (**8**), and hydroxytetrahydrorhombifoline (**10**) were identified within the minor components. Short-term (0.5 h) ultrasound-assisted extraction was demonstrated to be comparable with long-term (6 h) conventional extraction. 

All obtained fractions exhibited activity on the mycelial growth of the studied *F. oxysporum* strain (IC_50_ < 100 µg/mL). However, the extraction appeared to strongly impact the proportion of the most active constituents, with fraction UAE3 being the most active. After a statistical integration of the alkaloidal composition and bioactivity datasets, four compounds (i.e., **7**, **9**, **10**, and **12**) were more likely related to the mycelial growth inhibition since the extraction conditions appeared to affect the major components to the same extent. 13α-Hydroxylupanine (**12**) may represent a promising antifungal alkaloid to be included in further studies against the phytopathogen *F. oxysporum*.

## Figures and Tables

**Table 1:** Comparison of the chemical composition of alkaloidal fractions from *L. mirabilis* using conventional solvent and ultrasound-assisted extraction.

Compound	Relative Abundance (%) ^a^					
	CSE1	CSE2	CSE3	UAE1	UAE2	UAE3
**1**	0.3 ± 0.1 ^C^	0.7 ± 0.1 ^B^	0.7 ± 0.1 ^B^	0.3 ± 0.1 ^C^	1.1 ± 0.1 ^A^	0.5 ± 0.03 ^B,C^
**2**	74.3 ± 1.5 ^B^	80.4 ± 1.7 ^A^	80.6 ± 2.1 ^A^	79.6 ± 1.1 ^A^	74.4 ± 1.3 ^B^	64.7 ± 1.7 ^C^
**3**	-	0.8 ± 0.1 ^B^	0.8 ± 0.1 ^B^	0.8 ± 0.1 ^B^	1.3 ± 0.1 ^A^	-
**4**	-	-	0.6 ± 0.1 ^B^	0.2 ± 0.1 ^C^	1.2 ± 0.1 ^A^	0.7 ± 0.1 ^B^
**5**	-	-	-	-	0.4 ± 0.1 ^A^	-
**6**	-	0.5 ± 0.1 ^B^	0.03 ± 0.01 ^D^	0.3 ± 0.1 ^C^	0.7 ± 0.1 ^A^	-
**7**	1.6 ± 0.2 ^D^	3.0 ± 0.2 ^B,C^	2.7 ± 0.2 ^C^	3.5 ± 0.2 ^B^	3.6 ± 0.2 ^B^	4.9 ± 0.3 ^A^
**8**	0.4 ± 0.1 ^D^	1.5 ± 0.1 ^A,B^	1.8 ± 0.2 ^A^	1.0 ± 0.1 ^C^	1.0 ± 0.1 ^C^	1.4 ± 0.1 ^B^
**9**	-	0.3 ± 0.1 ^B^	0.2 ± 0.1 ^B^	0.3 ± 0.1 ^B^	0.3 ± 0.1 ^B^	0.6 ± 0.1 ^A^
**10**	0.6 ± 0.1 ^E^	0.9 ± 0.1 ^C,D^	0.8 ± 0.1 ^D,E^	1.2 ± 0.1 ^B^	1.1 ± 0.1 ^B,C^	1.6 ± 0.1 ^A^
**11**	4.5 ± 0.3 ^C^	7.9 ± 0.5 ^B^	7.7 ± 0.5 ^B^	10.0 ± 0.6 ^A^	9.9 ± 0.5 ^A^	10.4 ± 0.5 ^A^
**12**	-	0.4 ± 0.1 ^C^	0.8 ± 0.1 ^B^	-	1.0 ± 0.1 ^B^	1.7 ± 0.2 ^A^
**13**	-	0.4 ± 0.1 ^A,B^	0.2 ± 0.1 ^B,C^	0.4 ± 0.1 ^A,B^	0.2 ± 0.1 ^B,C^	0.6 ± 0.1 ^A^
**Total ^b^**	82.0 ± 2.1 ^B^	96.8 ± 2.7 ^A^	97.4 ± 2.3 ^A^	97.7 ± 1.2 ^A^	96.4 ± 1.5 ^A^	95.6 ± 1.8 ^A^

^a^ Results expressed as the mean values ± standard deviation (SD); ^b^ Total of nitrogen-containing compounds; - = not detected. CSE1-3: conventional solvent extraction for 2, 4, and 6 h, respectively; UAE1-3: ultrasound-assisted extraction for 0.5, 1 and 2 h, respectively. **1**: α-isosparteine; **2**: sparteine; **3**: β-isosparteine; **4** and **5**: unknown; **6**: ammodendrine; **7**: tetrahydrorhombifoline; **8**: 17-oxosparteine; **9**: α-isolupanine; **10**: hydroxytetrahydrorhombifoline; **11**: lupanine; **12**: 13α-hydroxylupanine; and **13**: docos-13-enamide. Different capital superscript letters across rows indicate significant differences among extractions per compound according to the post-hoc HSD Tukey’s testing (*p* < 0.05).

**Table 2:** Mycelial growth inhibition of alkaloidal fractions against *Fusarium oxysporum*.

	IC_50_ (µg/mL)	Confidence Interval (95%)
**CSE1**	98.5 ^E^	90.1–102.1
**CSE2**	91.3 ^DE^	84.3–93.7
**CSE3**	84.8 ^D^	80.0–87.3
**UAE1**	86.3 ^DE^	84.5–92.8
**UAE2**	62.3 ^C^	56.9–66.2
**UAE3**	33.5 ^B^	27.5–35.6
**P ^a^**	17.7 ^A^	15.5–20.6
**M ^a^**	11.9 ^A^	7.7–14.2

^a^ Positive controls: P = prochloraz; M = Mancozeb. CSE1-3: conventional solvent extraction for 2, 4, and 6 h, respectively; UAE1-3: ultrasound-assisted extraction for 0.5, 1, and 2 h, respectively. Different capital superscript letters across IC_50_ column indicate significant differences according to the Kruskal–Wallis test with Dunn′s multiple comparisons post-hoc test (*p* < 0.05).

## Data Availability

The authors confirm that the data supporting the findings of this study are available within the article and from the corresponding author upon request.

## Sample Availability

Samples are available from the authors upon request.

## Appendix A

**Table A1:** Identification of compounds from alkaloidal fractions of *L. mirabilis* leaves by GC-MS.

# ^a^	RT ^b^	RI ^c^	M^+ d^	Other MS Fragments ^d^									
**1**	15.87	1757	234(39)	98(100)	137(55)	136(36)	97(25)	110(23)	84(20)	193(17)	122(16)	134(15)	150(15)
**2**	16.73	1786	234(26)	137(100)	98(87)	136(42)	97(34)	193(33)	110(22)	122(19)	84(18)	150(15)	134(15)
**3**	16.93	1852	234(19)	137(100)	98(95)	136(43)	97(40)	193(28)	110(24)	122(21)	84(20)	134(18)	150(15)
**4**	17.24	1887	234(15)	98(100)	137(90)	134(46)	97(43)	122(26)	232(26)	110(24)	84(19)	150(17)	193(16)
**5**	17.62	1920	232(37)	98(100)	137(62)	97(44)	134(39)	84(29)	122(22)	110(19)	148(16)	193(15)	177(12)
**6**	17.75	1934	208(47)	165(100)	110(99)	136(88)	123(85)	191(78)	137(73)	94(69)	95(45)	84(40)	107(29)
**7**	19.75	2133	248(<1)	58(100)	207(87)	112(31)	108(16)	208(12)	55(18)	94(6)	84(4)	98(3)	-
**8**	19.99	2172	248(51)	97(100)	98(87)	110(73)	136(49)	220(35)	123(33)	137(28)	150(23)	134(22)	191(16)
**9**	20.36	2204	248(44)	136(100)	149(52)	98(38)	150(32)	97(31)	110(23)	137(22)	84(20)	134(18)	122(13)
**10**	20.63	2236	264(<1)	223(100)	58(68)	108(60)	96(20)	128(18)	224(14)	100(9)	110(6)	82(6)	-
**11**	20.88	2275	248(44)	136(100)	149(54)	150(36)	98(26)	110(22)	134(20)	137(15)	82(10)	219(7)	-
**12**	23.09	2531	264(30)	152(100)	134(50)	246(50)	165(38)	112(36)	148(26)	98(17)	122(15)	84(14)	207(12)
**13**	24.85	2623	337(3)	59(100)	72(67)	55(41)	43(28)	126(14)	86(10)	114(8)	140(4)	320(3)	294(2)

^a^ # = Compound numbering according to the retention time in the total ion chromatogram, whose names and structures are presented in Figure A1. ^b^ RT = retention time (min). ^c^ RI = Retention indexes (experimental). ^d^ Values in parenthesis constitute the ion’s relative intensity in the MS spectra.
